# An ANN model for the differential diagnosis of tuberculosis and sarcoidosis

**DOI:** 10.6026/97320630016539

**Published:** 2020-07-31

**Authors:** Mahalakshmi Vijayaraj, PA Abhinand, P Venkatesan, PK Ragunath

**Affiliations:** 1 Department of Bioinformatics, Faculty of Biomedical Sciences Sri Ramachandra Institute of Higher Education and Research (Deemed To Be University)

**Keywords:** Machine learning, Artificial Neural Network (ANN), Multi-layer perceptron (MLP), Pulmonary Tuberculosis (PTB), Sarcoidosis

## Abstract

Sarcoidosis is often misdiagnosed as tuberculosis and consequently mistreated owing to inherent limitations in histopathological and radiological presentations. It is known that the differential diagnosis of Tuberculosis and Sarcoidosis is often non-trivial and requires expertise and experience from clinicians. Therefore, it is of interest to describe a multilayer neural network model to differentiate pulmonary tuberculosis from Sarcoidosis using signal intensity data from blood transcriptional microarray. Genes that are significantly upregulated in Pulmonary Tuberculosis and Sarcoidosis in comparison with healthy controls were used in the model. The model classified Pulmonary Tuberculosis and Sarcoidosis with 95.8% accuracy. The model also helps to identify gene markers that are differentially upregulated in the two clinical conditions.

## Background

Tuberculosis (TB) is among the ten commonest causes of death worldwide[1-2]. Despite tremendous growth in TB control tools such as improved vaccines and novel drugs, there are still gaps in the development of fast and accurate diagnostic methods for TB.[3] Emergence of multidrug resistant tuberculosis (MDR TB) has become the biggest challenge to treat and spreading rapidly highlighting the adaptability of the pathogen.[4] Around 23% of the earth's inhabitants have latent TB. Across the under-developed world, especially in countries with a high population density and sub-optimal hygiene, tuberculosis remains a major life-threatening disease. The elimination of tuberculosis successfully has become a major threat to public health, and the process is further complicated with the rise in new strains of the tubercle bacillus that are resistant to conventional antibiotics[5]. Extrapulmonary tuberculosis (EPTB) occurs when TB infection develops outside the lungs. EPTB may also co-exist with Pulmonary Tuberculosis (PTB).About 15-20% of people may remain asymptomatic [6].

Sarcoidosis is a chronic, multisystemic granulomatous disease that involves abnormal collection of inflammatory cells forming lumps beginning in the lungs, skin or lymph nodes, which is of unknown aetiology and a close clinical mimic of TB. Sarcoidosis and PTB are both granulomatous, exhibit clinico-radiological presentations of great similarity, which makes differential diagnosis a huge challenge in countries with high prevalence. [7-10] A DNA microarray is a chip based technology consisting of microscopic DNA spots immobilised on to a solid surface, which measures expression levels of a large number of genes simultaneously or genotype multiple regions of a genome. [11] The co-existence of sarcoidosis and TB cannot be predicted since both the disease exhibit a similar kind of symptoms due to which the diseased gets the treatment for tuberculosis [12]. Radiological studies such as CT scanning and Chest X-rays are the only evident diagnosis applicable in differential diagnosis to check for non-caseating epithelial cell granuloma [13] Machine learning models for differential diagnosis in tuberculosis can prove a useful addition to human expertise. Machine learning is a subset of artificial intelligence based on statistics, data science and computational algorithms, which enables a computer to learn by drawing inferences from examples (learning) It can help in dealing with uncertainty involved in decision making [14]. Therefore, it is of interest to describe a multilayer neural network model to differentiate pulmonary tuberculosis from Sarcoidosis using signal intensity data from blood transcriptional microarray.

## Materials and Methods:

### Gene expression data:

An extensive systematic search of all literature pertaining to gene expression in Mycobacterium tuberculosis was performed by searching the Gene Expression Omnibus (GEO) datasets (as on April 2016) using the algorithm as given below. (Table 1)

G1 AND (("M" OR "m" OR "S" AND (H OR h))

G2 AND (("M" OR "m" OR "S" AND (H OR h))

G3 AND (("M" OR "m" OR "S" AND (H OR h))

Where,

G1 = Gene expression; G2 = Expression array; G3 = Microarray; M = Mycobacterium tuberculosis; m = TB; S = Sarcoidosis; H = Homo sapiens; h = human

The query lexicon was restricted to Homo sapiens so that only datasets from human studies would be included in the study. Only those studies in the English language were included for further analysis. On the basis of simple scoring algorithm the confidence of Literature mining is tested.

### Gene expression profiling:

Gene expression profiling of a data is the measure of the activity/expression of thousands of genes at once to analyse a wide range of cellular functions taking place in an organism. These profiles can distinguish an actively multiplying cell or to check the expression levels of cells that have reacted to the drug treatment. Many transcriptomics technologies are involved in measuring the expression levels of the genes, in a cell, which is expressed at a given time frame by quantifying the mRNA levels. This can encompass several thousand genes at the same time or sometimes even the entire genome. This can yield vital information on the activity, functions of the gene of our interest. [15] The GEO datasets with accession number GSE83456 annotated in GPL10558 platform were chosen by systematic text mining technique as described above. Gene expression profiling analysis of the chosen dataset using GEO2R was carried out. [16]

The dataset comprised of 61 healthy human controls, 47 humans with EPTB, 45 human with PTB and 49 humans with Sarcoid included. The present study EPTB was excluded as it was not complex in diagnosis and was not much significant and viable. The gene expression profiling values were log(base2) transformed and percentage shift normalization was performed. The fold change differences in gene expression between normal and disease samples were calculated for each gene separately. The mean differences between the samples were tested using ANOVA and the significance level is fixed at 4% level. A cut off value of 1.5 fold change was used to classify upregulated genes. [17-19] (Table 2)

### Multilayer Perceptron based Neural Network Model:

**Figure 1:**
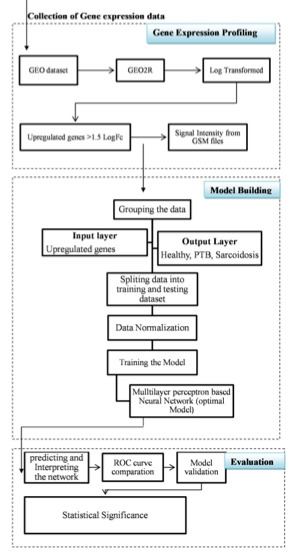
Research workflow is shown

Multilayer Perceptron to classify PTB, sarcoidosis, and Healthy controls were built by taking top upregulated genes from gene expression profiling of GSE83456 as covariates (input layer) and classifying into three groups (Healthy, PTB or Sarcoidosis as the output variable. All input variables were standardized and two-third was used for training and the remaining one-third for testing. For Normal, PTB and Sarcoidosis classification the input layer consists of 13 genes. The Multilayer Perceptron Neural Network with logistic activation function was used for three-way classification. Precision, recall and accuracy measures were used as validation measures. (Figure 1 and see Supplementary material S1 EXCEL file format)

### Evaluating the Goodness of the Predictive Values:

The Receiver Operating Curve (ROC) was employed for evaluating the goodness of the values predicted by the developed MLP models. In a ROC curve true positive rate (sensitivity) is plotted in function of the false positive rate (100-specificity) for different cut-off points of a parameter. Each point on the ROC curve represents a sensitivity/ specificity pair corresponding to a particular decision threshold. The area under the ROC curve (AUC) is a measure of how well a parameter can distinguish between two diagnostic groups (disease/ Normal).

In the current study the diagnostic groups were classified as Healthy, PTB, and Sarcoidosis. One of the reliable ways of evaluating the performance of the classifiers is through accuracy. The accuracy of a test is its ability to differentiate the diseased and healthy samples correctly. To estimate the accuracy of a test, we should calculate the proportion of true positive and true negative in all evaluated samples.

(i)True positives (TP)-correctly classified positive samples.

(ii)True Negative (TN)-correctly classified negative samples.

(iii)False positives (FP)-Misclassified negative samples.

(iv)False Negative (FN)-Misclassified positive samples.

ROC curves describe the relation between two indices namely (i) True Positive fraction (TPF) (ii) False Positive fraction (FPF).

ROC curves plot TPF (sensitivity) vs FPF (1-specificity) for every possible decision threshold imposed on the decision variable. Generally the Area under the ROC curve (AUC) is used as a measure of performance. One may be interested to compare TPF and FPF to learn the performance of two clinical diagnostic models where two ROC curves cross. [20] In statistics the positive predictive values (PPV) and negative predictive value (NPV) are proportional to positive and negative results and the diagnosis tests that are true positive and true negative results respectively. The PPV and NPV describe the performance of a predictive model.

In binary classification of statistical analysis, the F1 score is the harmonic mean of precision and sensitivity (recall) is a measure of a test's accuracy. P is ratio of the number of correct positive results and the number of all positive results returned by the classifier, and r is ratio of the number of correct positive results and the number of all relevant samples where all the samples are identified as positive. The F1 score is considered to be at its best when its value measures 1 (perfect precision and recall) and worst at 0. [21-22]

## Results:

**Figure 2:**
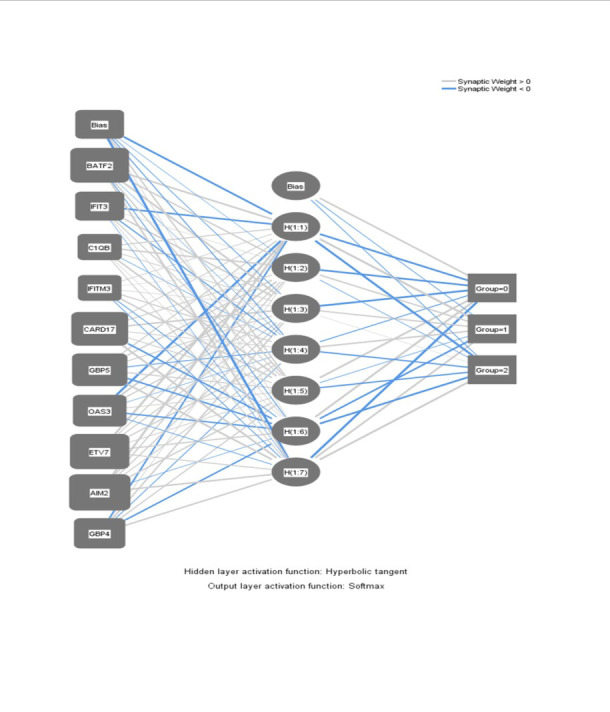
Multilayer Perceptron Neural Network to classify Healthy (0),Pulmonary Tuberculosis(1) ,Sarcoidosis(2)

Tuberculosis is classified into Pulmonary Tuberculosis, which affects the lungs, and Extra pulmonary, which affects the other parts of the body except for the lungs. Sarcoidosis is very similar to TB with common symptoms and clinical manifestations; it is often misdiagnosed and mistreated. The clinicoradiological evidence supported by laboratory finding and clinical acumen of the physician can help in the precise diagnosis of the two.[23] It is common to treat Sarcoidosis with empirical antitubercular therapy until the correct diagnosis is made, leading to drug toxicity and often-acute hepatic failure.[24] The current study is aimed at differential diagnosis of PTB, Sarcoidosis and Healthy samples using microarray data, which provides differentially expressed genes based on signal intensity to build an Artificial Intelligence (AI) based model for diagnosing active disease with healthy control. The study comprises of blood mRNA transcriptional response of tuberculosis (TB) patients to study the host immune response using microarray profiling. [25] Various experimental studies are collectively reanalyzed the publicly available datasets using different methodologies to identify resolutely differentially expressed genes which could distinguish active TB from healthy controls.The genes identified are potential candidates for biomarkers of active disease and additionally could provide valuable information regarding the immune and inflammatory response underlying TB pathogenesis. Machine learning-based MLP model seems to be very useful for the classification of sarcoidosis from PTB. The intensity of those genes whose expression levels are significantly different in PTB, Sarcoidosis and Normal healthy samples (P<0.05) were chosen as the input layer. Hyperbolic tangent was used as the activation function. Multilayer perceptron neural network model was built by prioritising the genes based on their weights in the neural network. The most discriminative genes associated with disease severity were 33. Though the blood transcripts revealed gene overlapping among sarcoidosis and tuberculosis, reapplication of machine learning algorithm were applied. The top10 genes were BATF2, IFIT3, C1QB, IFITM3, CARD17, GBP5, OAS3, ETV7, AIM2, and GBP4 that with the hidden layer weights >50% were chosen as the ideal candidates for building the model and which also serves as gene signatures to build multilayer perceptron neural network to classifying sarcoidosis from PTB. The neural network model was found to be capable of classifying Normal,PTB and Sarcoidosis with accuracy and the network was found to be capable of classifying with 95.8% accuracy (Figure 2) and Table 3) One of the ways of evaluating the classifier for its accuracy is through finding the ratio proportion of correctly classified total number of disease with the total number of healthy data.

**Figure 3:**
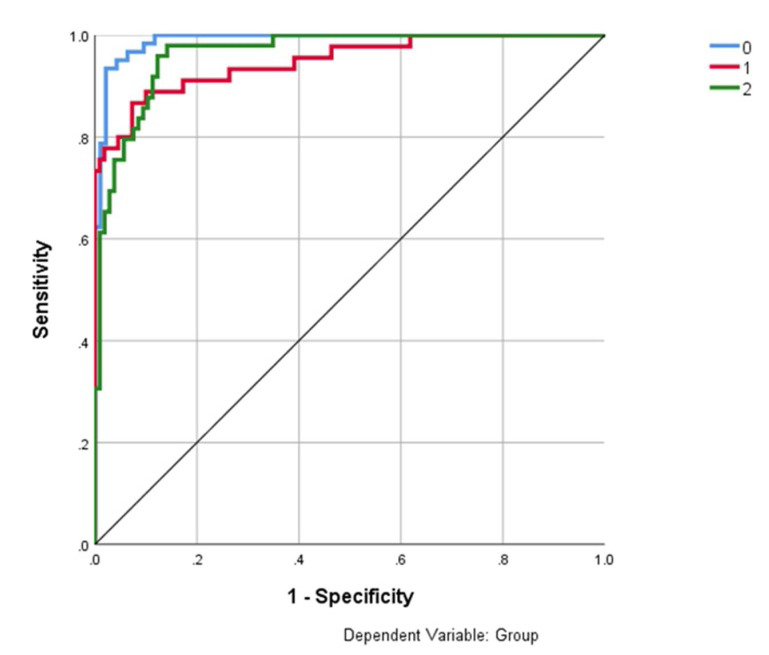
ROC curves comparing Sarcoidosis (Green-2), PTB (Red-1) and Healthy controls (Blue-0)

To compare different classification models, Receiver Operating Characteristic (ROC) curves were constructed and the Area under Curve (AUC) was calculated (PTB-0.949; Sarcoidosis-0.964) for the two models. The high AUC value is related to high accuracy rate. In ROC space X-axis is Specificity and Y-axis is sensitivity. At the standardized specific threshold, the model outputs specificity (100%) and sensitivity (95.8%), to draw a point in ROC space. All the points' Healthy control, PTB and Sarcoidosis converge into ROC curve. The ROC curve revealed highly significant classifying ability among the disease diagnosis. [26] Therefore both precision and recall values are based on the measure of relevance. The precision and recall for the present diagnostic model was found to be 95.92%. Precision gives the exactness or quality of the model, whereas recall gives the measure of completeness or quantity. The relationship between sensitivity and specificity to precision depends on the percentage of positive cases among the total number of the samples collected. Hence high precision means that more relevant results than irrelevant ones, while high recall means that most of the relevant results returned from an algorithm returned. The ROC curves of the two models are shown in (Figure 3) and see supplementary material S2 in EXCEL file format.

## Discussion:

The aim of the experiment was to (i) distinguish Sarcoidosis from PTB and healthy patients using machine-learning algorithm to classify the disease based on the signal intensity; (ii) possible gene signature were found to check for the overlapped genes whose expression level determines the severity of the disease to establish a easy diagnosis and provides an less opportunity to make erroneous diagnosis. Koth et al. analysed blood transcript using machine learning to look for the overlapping genes in sarcoidosis and PTB. By reapplication specific genes for sarcoidosis and PTB were identified. They analysed using three independent machine-learning algorithms:random forests, shrunken centroids [27], and elastic net were each algorithm gave a high sensitivity and specificity values to discriminate the sarcoidosis patients from PTB. Further Random forest was performed which showed the accuracy (87.9%) which is less compare to the current study.[28]

The samples collected from the diseased and/or control subjects were processed to obtain expression data at different times. The supervised machine learning classification methods are employed to discriminate the expression data of the patients having the disease or not having the disease. The testing training data may be obtained by any of the suitable machine learning classification methods which is typically used to determine the sensitivity, specificity and/or accuracy of the multilayer perceptron model which is capable of determining whether said data is indicative of pulmonary tuberculosis or Sarcoidosis. In our study the MLP model showed the generalised classification with an accuracy of 98.6%, specificity of 100%, and sensitivity of 95.83% when optimised for accuracy. There were several other methods as per the literature are compared further.

Yuanli Wu et al.[29] attempted an automatic classification of PTB and sarcoidosis using random forest by taking several features such as RBC deposit, Uric acid, haemoglobin, platelet, fasting plasma, etc. The random forest model showed a prediction accuracy of 85.33%. They also compared their prediction accuracy with other models such as Logistic regression (84.5%), Naive Bayes (85.06%), and Support vector classification (82.2%). The multilayer perceptron model described in the current study performed better than the diagnostic model proposed by Yuanli Wu et al. Showing 95.8%. While several machine learning models for automated diagnosis of tuberculosis using different methods such as logistic regression, deep learning, convoluted neural networks and support vectors. These models are based on different features like –MODS (microscopic Observation Drug Susceptibility) [30] Parameter from patient discharge reports, data from biochemical investigations, etc.[31-32] The current ANN models was unique in comparison with the existing statistical models built for similar purpose, as it was based on gene expression data and is capable of performing a three way classification between Sarcoidosis, Tuberculosis and Healthy controls and it also showed higher classification precision.

## Conclusions:

This study provides a quick diagnosis and accurate differential classification of Tuberculosis vs Sarcoidosis versus healthy tissue. Our model provides considerable scope to target therapy appropriately to patients with Sarcoidosis misdiagnosed as tuberculosis, and thus avoids mistreatment. Further studies are needed to validate the efficacy of the machine learning models in different populations. We describe a multilayer neural network model to differentiate pulmonary tuberculosis from Sarcoidosis using signal intensity data from blood transcriptional microarray.

## Figures and Tables

**Table 1:** Distribution of GEO datasets with different keywords

S.No	Keywords	No of Datasets
1	Gene Expression AND (("Mycobacterium tuberculosis" OR "TB" OR "Sarcoidosis "AND (Homo sapiens OR human))	1453
2	Expression array AND (("Mycobacterium tuberculosis" OR "TB" OR "Sarcoidosis" AND (Homo sapiens OR human))	221
3	Microarray AND (("Mycobacterium tuberculosis" OR "TB" OR "Sarcoidosis" AND (Homo sapiens OR human))	263
Total		1937

**Table 2:** Top 10% Upregulated overlapping genes in PTB, EPTB and Sarcoidosis.

Pulmonary tuberculosis(PTB)		Sarcoidosis	
Gene	Log2 FC	Gene	Log2 FC
AIM2	2.387	AIM2	1.463
ANKRD22	4.211	ANKRD22	3.356
ATF3	2.339	ATF3	1.708
BATF2	4.038	BATF2	3.052
C1QB	3.414	C1QB	2.302
CARD17	2.959	CARD17	2.018
CEACAM1	2.036	CEACAM1	1.451
EPSTI1	2.548	EPSTI1	1.874
ETV7	2.125	FCGR1A	2.555
FBXO6	1.997	FCGR1B	2.515
FCGR1A	3.304	GBP1	2.058
FCGR1B	3.162	GBP1P1	1.719
GBP1	2.486	GBP4	1.591
GBP1P1	2.359	GBP5	2.17
GBP4	1.784	GBP6	2.528
GBP5	2.567	IFI44L	2.102
GBP6	3.375	IFIT3	2.669
IFI44	2.12	IFITM3	1.791
IFI44L	2.851	LOC101930164	1.937
IFI6	2.059	OAS1	1.508
IFIT3	2.907	OAS3	1.649
IFITM3	2.639	P2RY14	2.17
LOC101930164	2.499	RSAD2	2.163
MYOF	1.707	RTP4	1.622
OAS1	1.921	SERPING1	3.226
OAS3	2.228	TIMM10	1.563
P2RY14	2.371		
RSAD2	2.921		
RTP4	2.229		
SCO2	1.957		
SERPING1	4.196		
TIMM10	2.201		
WARS	1.723		
XAF1	1.729		

**Table 3:** MLP-ANN Classification accuracy for Healthy controls PTB, Sarcoidosis.

Sample	Observed	Predicted			
		0 Healthy	1 PTB	2 Sarcoidosis	Percent Correct
Training	Healthy	34	1	1	94.40%
	PTB	2	25	6	75.80%
	sarcoidosis	4	5	29	76.30%
	Overall Percent	37.40%	29.00%	33.60%	82.20%
Testing	Healthy	24	0	1	96.00%
	PTB	0	11	1	91.70%
	sarcoidosis	0	0	11	100.00%
	Overall Percent	50.00%	22.90%	27.10%	95.80%
